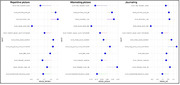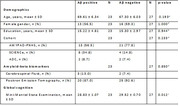# Multi‐day at‐home assessments of speech acoustics in Dutch cognitively normal adults with‐and without AD pathology

**DOI:** 10.1002/alz.087304

**Published:** 2025-01-03

**Authors:** Rosanne L. van den Berg, Casper de Boer, Marissa D. Zwan, Jessica Robin, William Simpson, Roos J Jutten, Mark A. Dubbelman, Frederik Barkhof, Lyduine E. Collij, Argonde C. van Harten, Lisa‐Marie Schlüter, Charlotte Teunissen, Wiesje M. van der Flier, Sietske A.M. Sikkes

**Affiliations:** ^1^ Alzheimer Center Amsterdam, Neurology, Vrije Universiteit Amsterdam, Amsterdam UMC location VUmc, Amsterdam Netherlands; ^2^ Amsterdam Neuroscience, Neurodegeneration, Amsterdam Netherlands; ^3^ Faculty of Behavioural and Movement Sciences, Clinical Developmental Psychology & Clinical Neuropsychology, Vrije Universiteit Amsterdam, Amsterdam Netherlands; ^4^ Winterlight Labs (Cambridge Cognition), Toronto, ON Canada; ^5^ Winterlight Labs, Toronto, ON Canada; ^6^ Massachusetts General Hospital, Harvard Medical School, Boston, MA USA; ^7^ Harvard Medical School, Boston, MA USA; ^8^ Amsterdam UMC, Amsterdam Netherlands; ^9^ Department of Radiology and Nuclear Medicine, Vrije Universiteit Amsterdam, Amsterdam University Medical Center, location VUmc, Amsterdam Netherlands; ^10^ Alzheimer Center Amsterdam, Neurology, Vrije Universiteit Amsterdam, Amsterdam UMC, Amsterdam Netherlands

## Abstract

**Background:**

Automated analysis of natural speech is emerging as a promising digital biomarker of Alzheimer’s disease (AD). As speech is a complex process, relying on multiple interacting cognitive functions, fine‐grained analysis of speech may have the potential to capture subtle cognitive deficits in the very early stages of AD. Here, we examined the association between amyloid‐beta (Aβ) pathology and acoustic speech characteristics in a group of cognitively normal Dutch adults.

**Method:**

We included 50 cognitively normal (n = 23 Aβ‐positive, 46%) older adults from three clinical cohorts at the Alzheimer Center Amsterdam (Table 1). Aβ‐status was based on local cut‐offs for Aβ_1‐42_‐concentrations in cerebrospinal‐fluid or visual inspection of amyloid positron emission tomography (PET)‐imaging. In a five day remote burst assessment, seventeen tablet‐based tasks (picture description, journal‐prompt storytelling, verbal‐fluency) were administered, with a daily administration time of approximately 10 minutes. Various acoustic features, including silent pauses, pause‐to‐word‐ratio, and perceived loudness, were extracted from the voice recordings. Mean burst scores were calculated over the five‐day period. The association between Aβ‐pathology and acoustic features was investigated using linear regression models separately for each subtask.

**Results:**

Pause‐to‐word ratio was higher in Aβ‐positive individuals in the repetitive picture description subtask (β = ‐0.05, p = 0.04) and the journaling subtask (*β* = ‐0.07, *p =* 0.03) compared to the Aβ‐negative individuals. Although statistical significance between groups was not reached for other acoustic features, a pattern was observed that the Aβ‐positive group consistently displayed more medium pauses, longer audio duration, more local jitter, higher intensity variance, a higher fundamental frequency, and fewer long pauses in all multi‐day subtasks (Figure 1).

**Conclusion:**

Our results demonstrated that Aβ‐pathology was associated with more pauses during free speech in cognitively healthy adults. This supports the notion that remote multi‐day speech assessments have the potential to monitor cognitive changes, for example in observational studies and decentralized clinical trials in preclinical AD. Next steps include examining the association between Aβ‐pathology and linguistic features using the same sample.